# Cavitation-induced traumatic cerebral contusion and intracerebral hemorrhage in the rat brain by using an off-the-shelf clinical shockwave device

**DOI:** 10.1038/s41598-019-52117-5

**Published:** 2019-10-30

**Authors:** Abel Po-Hao Huang, Dar-Ming Lai, Yi-Hua Hsu, Yi Kung, Chiang Lan, Chia-Shan Yeh, Hsin-Han Tsai, Chih-Feng Lin, Wen-Shiang Chen

**Affiliations:** 1 Department of Surgery, National Taiwan University Hospital and College of Medicine, Taipei, Taiwan; 2 Department of Physical Medicine and Rehabilitation, National Taiwan University Hospital and College of Medicine, Taipei, Taiwan; 3 Department of Otolaryngology, National Taiwan University Hospital and College of Medicine, Taipei, Taiwan

**Keywords:** Diseases of the nervous system, Experimental models of disease

## Abstract

Traumatic cerebral contusion and intracerebral hemorrhages (ICH) commonly result from traumatic brain injury and are associated with high morbidity and mortality rates. Current animal models require craniotomy and provide less control over injury severity. This study proposes a highly reproducible and controllable traumatic contusion and ICH model using non-invasive extracorporeal shockwaves (ESWs). Rat heads were exposed to ESWs generated by an off-the-shelf clinical device plus intravenous injection of microbubbles to enhance the cavitation effect for non-invasive induction of injury. Results indicate that injury severity can be effectively adjusted by using different ESW parameters. Moreover, the location or depth of injury can be purposefully determined by changing the focus of the concave ESW probe. Traumatic contusion and ICH were confirmed by H&E staining. Interestingly, the numbers of TUNEL-positive cells (apoptotic cell death) peaked one day after ESW exposure, while Iba1-positive cells (reactive microglia) and GFAP-positive cells (astrogliosis) respectively peaked seven and fourteen days after exposure. Cytokine assay showed significantly increased expressions of IL-1β, IL-6, and TNF-α. The extent of brain edema was characterized with magnetic resonance imaging. Conclusively, the proposed non-invasive and highly reproducible preclinical model effectively simulates the mechanism of closed head injury and provides focused traumatic contusion and ICH.

## Introduction

Traumatic brain injury (TBI) is a heterogenous phenomenon, and a variety of animal models have been developed to simulate different injury mechanisms^1,2^. Traumatic cerebral contusion and intracerebral hemorrhage (ICH), collectively called traumatic parenchymal lesions, have been reported to occur in more than 30 percent of severe TBI cases and have been associated with significant morbidity and mortality rates^3^. They are characterized by dying neurons showing cytoplasmic shrinkage and nuclear pyknosis, along with varying degrees of hemorrhage from ruptured capillaries^4^. Since the mechanisms underlying traumatic parenchymal lesions are complicated, it is difficult to develop proper models and effective therapies^5^.

Among the developed models, the controlled cortical impact (CCI) TBI model has been widely used to study traumatic contusions and ICH, in which brain trauma is produced by applying a pneumatic impactor to the exposed brain via craniotomy^6^. Unfortunately, this approach is subject to significant drawbacks. First, the craniotomy itself produces brain injury, damaging normal brain tissue, activating microglia, and producing evidence of astrogliosis indicating brain injury. Such injury has been found to be sufficient to produce behavioral deficits^7^. Secondly, CCI injury is widespread, including acute cortical, hippocampal and thalamic degeneration^8^. Finally, the CCI model may cause widely varying degrees of injury severity^1,2^. The drawbacks of the CCI model are also seen in other TBI models, such as the fluid percussion injury model and the weight drop model^1,2^. Therefore, there is a need to create a consistent and repeatable animal model that simulates traumatic contusion and ICH at focused brain areas while obviating the need for craniotomy.

In recent years, it has been suggested that TBI and ICH share barotrauma from pressure waves that propagate through the brain parenchyma as a common injury mechanism^5^. Shockwave is a strong form of pressure wave that produces cavitation when propagated through a fluid^9^, which *in vivo* disrupts the integrity of the tight junctions of the brain capillaries, and thus may be ideal for creating focal brain injuries. Kabu *et al*. found that extracorporeal shockwaves (ESWs) induced blood-brain barrier (BBB) opening accompanied by edema formation in rat brains^10^. Nakagawa *et al*.^11^, Hatano *et al*.^12^ and Takeuchi *et al*.^13^ showed ICH and contusion-like brain injury around the hematoma in the rat brain after ESW application. Recently, Liu *et al*.^14^ demonstrated that ESWs induced apoptotic cell death as well as microglial activation. However, there is still room for methodological improvement. Firstly, the obtained brain injuries were diffuse and inconsistent when keeping the animal skull intact^10^. Secondly, craniotomy was needed to increase the susceptibility of brain tissue to ESWs for focal injury^11–13^. Moreover, these studies used home-made instruments to generate ESWs, increasing the difficulty for reproducibility in other labs^11–14^.

In the present study, we evaluated the feasibility of using a clinically available and off-the-shelf ESW device (Richard Wolf’s PiezoWave) as the general platform to preclinically create brain contusions and ICH. Our recent work has shown that this device can disrupt the BBB based on cavitation and without craniotomy^15^. We further used microbubbles to enhance the ESW-induced cavitation in order to mimic the barotrauma mechanisms. By coupling the use of a concave ESW probe, our results demonstrated focal and highly reproducible contusion and ICH in the animal brain with intact skull after applying ESWs plus microbubbles. Moreover, our results suggested that the severity of injuries can be readily controlled by adjusting the ESW parameters provided by the device. For characterization of the barotrauma-related injuries, we also applied histology and magnetic resonance imaging (MRI) to delineate the temporal changes of edema following BBB disruption, apoptosis, neuroinflammation and astrogliosis, the typical abnormalities in TBI. Conclusively, the proposed non-invasive and highly reproducible preclinical model effectively simulates the barotrauma mechanisms and provides focused traumatic contusion and ICH.

## Materials and Methods

### Animals

A total of 114 adult male Sprague Dawley rats (8–10 weeks old; National Laboratory Animal Center, Taipei, Taiwan) were used. The rats were housed in a pathogen-free environment with a 12:12-hour light:dark cycle and controlled humidity and temperature, with *ad libitum* access to food and water. All protocols applied in the experiments were approved by the Institute of Animal Care and Utilization Committee at Academia Sinica, Taipei, Taiwan. Methods applied in the animal study were approved by the ethics committee of the Laboratory Animal Center at National Taiwan University College of Medicine (approval No. 20170072) and carried out in accordance with the relevant guidelines and regulations.

### ESW application

**Figure 1:**
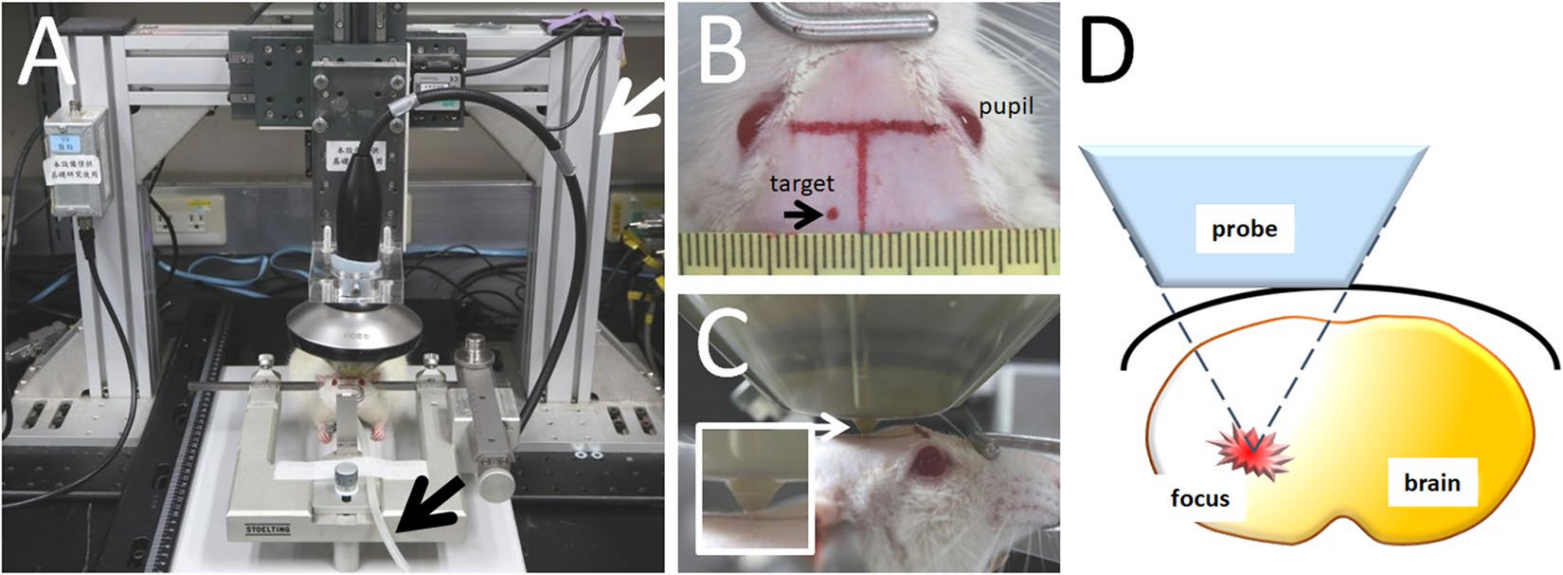
ESW application setup. (**A**) Device setup. The ESW probe was fixed on a turned U-shaped positioning platform (white arrow) and positioned over the shaved rat head fixed on a sterotaxic frame. The rat was anesthetized with 2% Forane delivered via a hose (black arrow). (**B**) The red spot labeled on the scalp indicates the target of the concave ESW probe. (**C**) A close-up photo showing the bud (arrow; i.e., probe center) on the bottom of the gel pad positioned over the labeled spot (i.e., target). (**D**) Depiction of the exact position of the ESW focal point in the rat brain^44^, i.e., 5 mm below the gel bud.

A PiezoWave ESW device was purchased from Richard Wolf GmbH (Knittlingen, Germany). Figure 1 shows the setup of the ESW device and the probe (F10 G4) positioning platform, which was home-made and designed to improve experiment reproducibility and accuracy in applying ESWs to the target brain area. A LabView-based computer program was used to minimize human error^15^, with the localization accuracy of the positioning operational program reaching a precision of 0.1 mm. The concave ESW probe was coupled with a gel pad to maintain a constant ESW focus 5 mm below the probe tip.

Animals were initially anesthetized with 5% Forane (i.e. Isoflurane; Aesica Queenborough Ltd., Queenborough, UK) in oxygen at a flow rate of 5 L/min, and maintained with 2% Forane in oxygen at a flow rate of 1 L/min throughout the experiment. The femoral vein was then cannulated with a polyethylene catheter (PE-50; Becton Dickinson, CA, USA) for intravenous injection of dye and contrast agent. After surgery, the heads of the anesthetized rats were shaved to expose the scalp and then firmly fixed on a sterotaxic frame (Stoelting Co., IL, USA) with one incisor bar and two ear bars. A spot was labeled with oil pen on the scalp at 3 mm laterally to the head midline and at 9 mm posteriorly to the pupil (Fig. 1), which was found to be almost equal to 0.5 mm anterior to the bregma on the skull in a pilot study (data not shown). The center of the ESW probe, together with a gel pad, was positioned over the spot labeled on the scalp to produce an energy focus 5 mm beneath the scalp surface. Ultrasound coupling gel (CG955, sonic resistance: 1.55 ± 0.05 MRayl; Ceyotek, Chiayi City, Taiwan) was inserted between the probe and the gel pad, and between the gel pad and the scalp. Prior to ESW application, Evans Blue dye (EB; 3% powder in saline; 0.1 mL/100 grams of body weight; MilliporeSigma, MO, USA) was injected to reveal the location and extent of BBB opening in the rat brains^15^. Since EB cannot cross an intact BBB, the EB found in the brain can be considered to be a quick indicator of the ESW effect on the brain. Following EB injection, SonoVue, an ultrasound contrast agent or microbubble (0.02 mL/100 grams^16,17^, similar to clinical dose; Diagnostics Inc., Milano, Italy) was injected to enhance the ESW-induced cavitation effect.

About 20 seconds after SonoVue injection, ESWs were applied to the rat brains. The rats were divided into four groups: **(1)** ESWs were applied to rats (N = 50) under 25 different conditions to evaluate the potential for inducing traumatic contusions and ICH, including varying ESW iterations (1, 2, 4, 8, 16; pulse repetition frequency (PRF) 1 Hz) at various intensity levels (1, 2, 4, 8, 16). The intensity levels for each parameter are shown in Table 1. The brains were collected 24 hours after transcardial perfusion with 4% paraformaldehyde solution for further histology. **(2)** Rats with mild or severe TBI at different time points were prepared to temporally analyze the pathological effects of ESWs on the rat brain. For mild TBI, one ESW was applied at intensity level 2, while for severe TBI, eight ESWs were applied at intensity level 2. The brains were collected at one day (N = 6), three days (N = 6), one week (N = 6), and two weeks (N = 6) for histology. **(3)** To study the expressions of inflammatory cytokines in the TBI rat brain, rats with severe TBI (N = 6; N = 6 for control) were prepared and their brain tissue was collected three days after ESW exposure for further cytokine assays. **(4)** To relate the pathological findings to imaging studies, the brains of rats with mild or severe TBI (N = 2, respectively) were imaged using a magnetic resonance imaging (MRI) scanner.

**Table 1:** Major ESW parameters of the intensity levels.

Intensity level	1	2	4	8	16
Negative peak pressure (MPa)	−7.3	−7.92	−9.17	−10.92	−14.21
Positive peak pressure (MPa)	11.8	13.66	17.37	23.1	48.1
Energy flux density (Total) (mJ/mm^2^)	0.1	0.13	0.18	0.27	0.6

### Histological examinations

The collected brains were embedded in paraffin, sectioned at 5 μm, and subjected to H&E (for general pathology, such as hemorrhages), TUNEL assay (for apoptosis), Iba1 immunostaining (for activated microglia), or GFAP immunostaining (for reactive astrocytes). Slides were scanned using the Motic EASY SCAN PRO Pathology Slide Scanner (MEYER INSTRUMENTS, INC., TX, USA) and analyzed with its software, DSALite. 0.7X magnification was used to show the whole brain section, while 15X magnification was applied to characterize the damaged tissue and measure the stained cells. To estimate the number of immunopositive cells in the ipsilateral striatum, a region of interest (2 mm width and 1 mm height) at the center of the striatum (about 5 mm depth beneath the cortical surface), i.e., the ESW focus, was photographed for each section. The stained cells in the obtained image were counted by a histologist blind to experimental conditions.

### Cytokine assays

The brain tissues were analyzed for inflammatory cytokine levels using the Bio-Plex cytokine assay system (Bio-Rad Laboratories, CA, USA) to quantify the concentrations of IL-1β, IL-6, TNF-α (Rat 9-plex kit) as the representative cytokines^18^. Collected tissues were processed and analyzed according to the instructions of the manufacturer using a Bio-Plex 200 system (Bio-Rad).

### MRI

MR images were obtained at 7 Tesla PharmaScan 70/16 (Bruker, Germany) with an active shielding gradient at 300 mT/m in 80 μs, with rats in a prone position. To show the *in vivo* rat brain anatomy, images with a field of view (FOV) of 2.56 cm, a slice thickness of 1 mm and a matrix size of 256 × 128 were acquired. Images were zero-filled to 256 × 256, resulting in an in-plane resolution of 100 μm × 100 μm. For T2WI, a fast spin-echo sequence was used (TR = 4000 ms, echo train length = 8, effective TE = 70 ms, NEX = 4). During scanning, body temperature was maintained at 37 °C using a warm-water blanket, and respiratory rate was monitored and maintained at 40~50 breaths per minute by altering the Forane levels.

### Statistics

All data are expressed as mean ± standard error (SE). All statistical evaluations were carried out with one-way ANOVA and post-hoc analysis. A p-value less than 0.05 was considered significant.

## Results

### Gross observation

**Figure 2:**
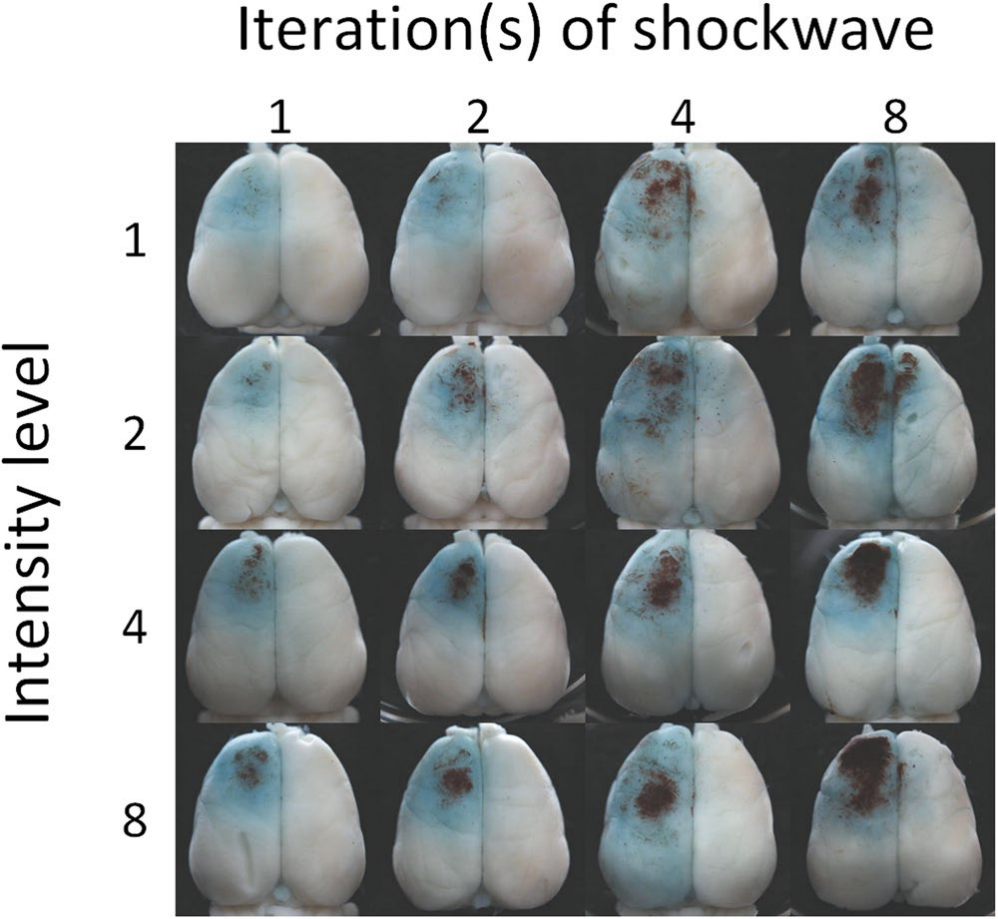
Gross observation of rat brains following ESW exposure. The blue and dark brown signals indicate EB dye leaked from the disrupted BBB and hemorrhaged on the brain surface. These gross findings are identical to the surgical findings in patients with traumatic contusion and ICH.

Rats exposed to the maximum number of ESW iterations (16) or intensity (16) showed high rates of mortality (more than 30%) while the surviving rats showed significant behavioral deficits after ESW exposure. Such ESW parameters were excluded from the study due to animal welfare considerations. The brains of the remaining rats were removed and photographed 24 hours following ESW exposure. As demonstrated in Fig. 2, ESW application always caused pathologic changes in the brain, regardless of the conditions applied. Visible damage included BBB disruption (indicated by EB) and hemorrhages on the brain surface, mainly found on the anterior part of the left hemisphere. Brains with low ESW exposure (i.e., one iteration and intensity level 1) showed very tiny hemorrhages in the area with BBB disruption. Interestingly, the hematoma size gradually increased with the number of iterations or intensity level. Most significantly, hematoma was found in brains with high exposure at eight iterations and intensity level 8. In the following experiments, mild injury (showing distinct small hemorrhages) was defined and induced by a single exposure at intensity level 2, while eight iterations at intensity level 2 produced severe injury (showing intact hematoma on the brain surface)^19,20^.

### Histopathological changes

**Figure 3:**
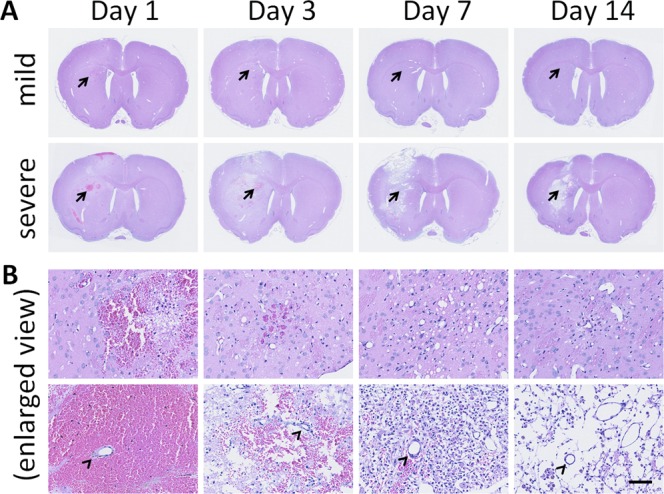
Histopathological changes in rat brains two weeks after ESW-induced focal injury. (**A**) H&E-stained sections of brain tissues, chosen from six candidates at each time point. Upper panel, mildly injured brain sections; lower panel, severely injured brain sections; arrows, areas enlarged and shown in (**B**). (**B**) Enlarged views (scale bar = 100 µm). Arrowheads, intact small vessels in the injured tissues.

Slide scanning results found the H&E-stained brain tissue of the non-exposed/contralateral side showed normal morphology and regular neuron arrangements with clearly visible nuclei. The ESW -induced TBI was focal, since the lesions were mainly found in the cortex and striatum (Fig. 3). The severely injured brain sections showed obvious swelling and midline shift on Days 1 and 3, followed by atrophy on Day 14 on the ipsilateral side (Fig. 3A). By contrast, mildly injured brain sections showed neither swelling/midline shift nor atrophy. The histopathological changes in the mildly injured tissue were demonstrated in the enlarged views (Fig. 3B). On Day 1, several contusion-like lesions were found in the cortex and striatum, accompanied by small hemorrhages. From Day 3, these small hematoma resolved gradually, followed by the formation of small vacuoles on Day 7 and gliosis on Days 7 and 14. Enlarged images showed significant abnormalities in the severely injured tissue (Fig. 3B). On Day 1, large hemorrhages appeared along with contusions and small hemorrhages, especially in the striatum. Interestingly, the small vessels in the large hematoma were likely intact (arrowheads in Fig. 3B), suggesting bleeding from damaged capillaries instead of small and/or large vessels. On Day 3, these large hematoma gradually resolved, accompanied by the formation of vacuoles. On Day 7, the vacuoles were filled by numerous cells which may proliferate microglia and/or infiltrate macrophages. The severely injured tissue eventually became a fluid-filled cavity on Day 14 as a result of brain atrophy.

### Apoptosis, inflammation, astrogliosis, and edema following ESW exposure

**Figure 4:**
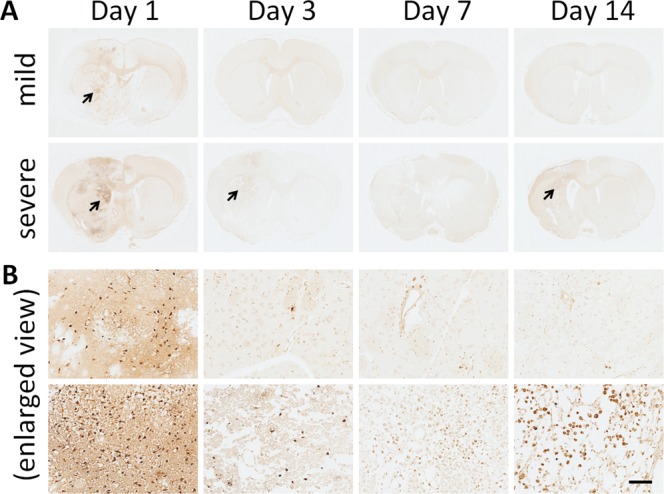
TUNEL assay of apoptotic cell death in brain tissues after ESW exposure. (**A**) TUNEL-stained sections of brain tissues, chosen from six candidates at each time point. Arrows, the areas with obvious TUNEL signals. (**B**) Enlarged detailed section views (scale bar = 100 µm).

TUNEL assay was performed to further investigate the effects of ESW exposure in the brain. As shown in Fig. 4A, the TUNEL signals in the sections were found to be localized in the ipsilateral side, without affecting the contralateral side or ipsilateral hippocampal or thalamic region, suggesting the occurrence of apoptotic cell death due to ESW-induced focal injury. In Fig. 4B, the enlarged views of the stained sections revealed numerous TUNEL-positive cells in the mildly and severely injured tissues on Day 1. From Day 3, the TUNEL-positive cells in the mildly injured tissues gradually disappeared. Interestingly, the existence of TUNEL-positive cells in the severely injured tissue became ambiguous from Day 3 and then became obvious again on Day 14.

**Figure 5:**
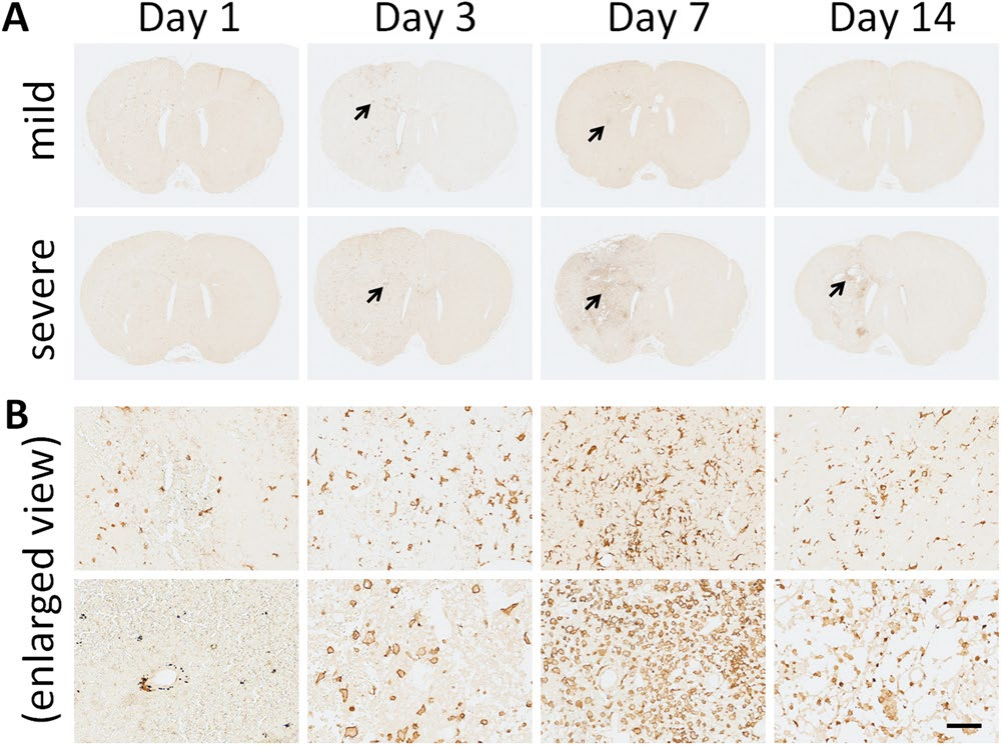
Iba1 immunostaining of microglial changes in brain tissue after ESW exposure. (**A**) Iba1-stained sections of brain tissues, chosen from six candidates at each time point. Arrows, the areas with obvious Iba1 signals. (**B**) Enlarged detailed section views (scale bar = 100 µm).

Iba1 immunostaining was performed to assess microglial activation and proliferation as an indicator of brain inflammation. As shown in Fig. 5A, the Iba1 signals in the sections were found to be localized in the ipsilateral side, not in the contralateral side or ipsilateral hippocampal or thalamic region, suggesting inflammation in the brain due to ESW-induced focal injury. In Fig. 5B, the enlarged views of the stained sections revealed microglial proliferation in either mildly or severely injured tissue from Day 3 in comparison with Day 1. On Day 14, microglial proliferation stopped. Interestingly, the morphology of the affected microglia in severely injured tissues was different from that in mildly injured tissues. On Day 1, the severely-affected microglia shrank significantly, probably reflecting the lethal impact of severe ESWs on the neural cells. On Days 3 and 7, the severely-affected microglia assumed a large, ameboid shape, a morphology typical of reactive microglia. The severely-affected microglia then shrunk again, which may be related to the increased TUNEL- positive cells on Day 14.

**Figure 6:**
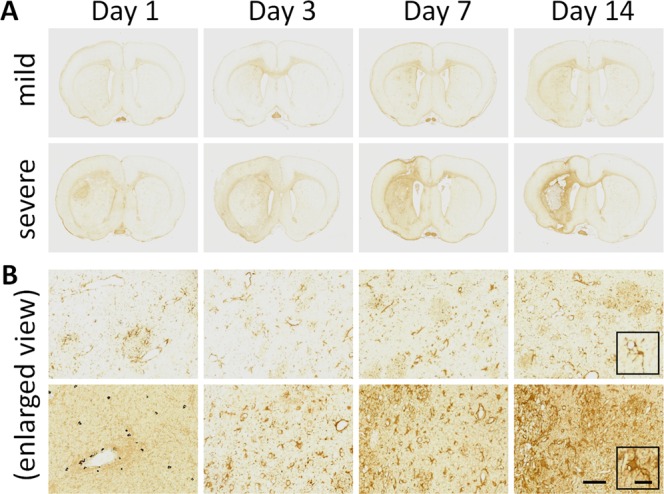
GFAP immunostaining of astrogliosis in brain tissue after ESW exposure. (**A**) GFAP-stained sections of brain tissues, chosen from six candidates at each time point. (**B**) Enlarged detailed section views (scale bar = 100 µm). The typical cells were further enlarged in the black squares (scale bar = 40 µm).

GFAP immunostaining was also performed to detect the activation and proliferation of astrocytes in the damaged brain. As shown in Fig. 6A, the GFAP signals were mainly found in the ipsilateral striatum after ESWs. In Fig. 6B, the enlarged views of the stained sections revealed a gradually increased GFAP signals, particularly in severely injured tissue. Interestingly, the morphology of the GFAP-positive astrocytes in severely injured tissues was different from that in mildly injured tissues. As shown in the further enlarged views, astrocytes in the mildly injured striatum had small round cell bodies and long and slim processes. By contrast, astrocytes in the severely injured striatum exhibited hypertrophied morphology with retracted processes.

**Figure 7:**
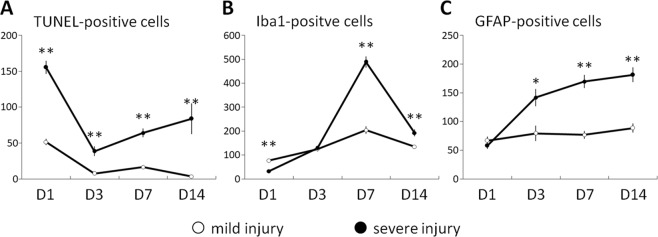
Time course of TUNEL-, Iba1-, and GFAP-positive cells in ESW-induced focal injury. (**A**) TUNEL-positive apoptotic cells. (**B**) Iba1-postive microglial cells. (**C**) GFAP-positive astrocytes. Six rats were sampled at each time point. Severe injury group (solid circle) vs. mild injury group (open circle), *p < 0.05, **p < 0.01.

The numbers of TUNEL-, Iba1- and GFAP-positive cells were quantified and charted. As shown in Fig. 7, the number of TUNEL-positive cells peaked on Day 1, suggesting that ESW exposure immediately caused apoptotic cell death. Moreover, the number of TUNEL-positive cells in cases of severe injury was three times that found in mild injury cases, reflecting the ESW intensity. On Day 3, the number of TUNEL-positive cells decreased. Thereafter, the number of TUNEL-positive cells gradually increased in severe injury cases. In contrast to TUNEL-positive cells, the numbers of Iba1-positive cells peaked on Day 7, suggesting that the ESWs promoted neuroinflammation at later stages. Interestingly, the number of Iba1-positive cells in severe injury cases was significantly lower than that in mild injury cases on Day 1 but significantly higher on Day 7, possible due to strong ESWs causing severe damage to neural cells, producing neuroinflammation and secondary injury. Unlike TUNEL- and Iba1-positive cells, the numbers of GFAP-positive cells peak on Day 14, probably reflecting the glial scar formation after significant loss of neurons.

**Figure 8:**
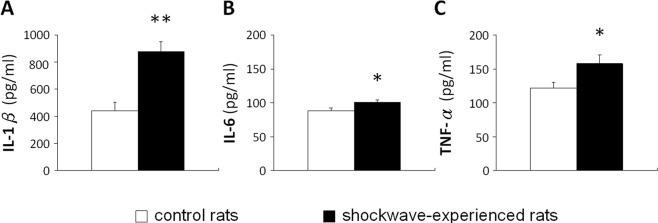
Expression of inflammatory cytokines after ESW exposure at severe condition. (**A**) IL-1β. (**B**) IL-6. (**C**) TNF-α. Six rats were sampled on Day 3 for each condition. ESW group (solid bar) vs. control group (open bar), *p < 0.05, **p < 0.01.

The ESW-induced neuroinflammation was also characterized by evaluating the expressions of inflammatory cytokines, including IL-1β, IL-6, and TNF-α. The brain tissues for cytokine assay were collected on Day 3, in which microglia started to show a reactive morphology (Fig. 5B). As shown in Fig. 8, the expressions of three representative cytokines extracted from injured tissues all were significantly higher than those from controls.

**Figure 9:**
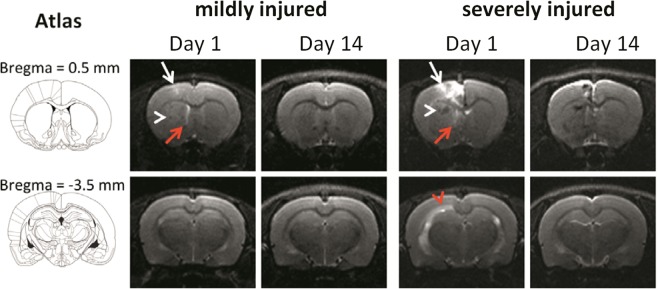
T2WI showing the different patterns of brain edema, ICH, and enlarged lateral ventricle in the mildly and severely injured brains. Images were acquired at the level of Bregma 0.5 mm (upper) or −3.5 mm (lower). White arrow, hyperintensity related to brain edema; white arrowhead, hypointensity related to ICH; red arrow, enlarged lateral ventricle; red arrowhead, hyperintensity related to the further enlargement of lateral ventricle.

As shown in Fig. 2, ESW exposure caused BBB disruption on Day 1, raising the possibility of brain edema, a typical pathology following contusion. MRI was performed to relate the histopathological findings to medical images. Based on T2WI, Fig. 9 shows abnormal hyperintensities in the ESW-exposed side on Day 1, particularly in the cortex, suggesting the formation of edema. Abnormal hypointensities were mainly found in the striatum, suggesting the presence of ICH. The patterns of abnormal T2WI signals were close to those of the brain injuries shown in Fig. 3A. T2WI also revealed enlargement of the lateral ventricle. In mildly injured brains, an enlarged lateral ventricle was only found beside the striatum at the level of Bregma 0.5 mm, suggesting the focusing ability of the ESW. Abnormal hyperintensities were also found in the external capsule beside the hippocampus at the level of Bregma −3.5 mm, suggesting the further enlargement of lateral ventricle in the hippocampal area due to stronger ESW exposure. Abnormal MRI signals decreased from Day 1 to Day 14.

## Discussion

### Advantages of our model

In recent years, ultrasound has been widely used to induce localized BBB opening^21^. In comparison with high intensity focus ultrasound (HIFU)^22^, ESW-induced cavitation also potentially disrupts the BBB through similar mechanisms^9^. However, HIFU may produce thermal damage to brain tissues during sonication (data not shown), and its transducing depths are limited at higher frequencies. By contrast, ESWs have a shorter pulse duration and thus include low-frequency acoustic wave components which may better transmit through the skull to deep brain tissue than HIFU^15,23^. Thus, the present study used a commercially available, off-the-shelf device as the general platform to generate ESWs, therefore obviating the need for building complex ESW devices as seen in earlier blast-associated models^10–14^. Furthermore, the patterns of the resultant brain injuries can be readily replicated and examined at different labs.

Blast has been used to produce ESW-associated TBI, which often leads to less controllable (i.e., diffuse and inconsistent) brain injury^1,2,10^. Compared to the blast-induced models, the proposed approach uses focused piezo technology to generate ESWs with microbubbles (SonoVue) to create focal traumatic contusions and ICH. Using the concave probe plus microbubbles to enhance the cavitation effects, we were able to transform the diffuse injury model into a focal model. Unlike other conventional TBI models such as CCI^8^, the proposed approach showed no hippocampal or thalamic lesions, suggesting that our model is more focal. Initially, we attempted to create this focal contusion and ICH model using ESWs without microbubbles, but increased ESW intensity and iterations were needed to create the same severity and the lesions were more diffuse. More importantly, for unknown reasons, the injury severity and type varied despite consistent parameter settings (data not shown). Therefore, we eventually used microbubbles to enhance the cavitation effects.

Commercially available extracorporeal shockwave therapy (ESWT) systems are currently widely used in hospital or clinical settings for treatment of tendinosis calcarea and kidney stone^24^. These systems are easy to set up, operate, and maintain. Since each animal takes less than ten minutes to prepare, multiple injured brain models can be created quickly. Divani *et al*. used anther clinical device, namely the Medstone STS-T system (Medstone International, Inc., Austin, TX) to generate ESWs and create blast-induced TBI models^25^. This system generates ESWs using electrohydraulic pressure from an electric spark-gap in a water reservoir and is then propagated toward the focal point via an ellipsoidal reflector. Clinical applications have centered on treatment of kidney stones. These two systems both produce ESWs, but the Medstone STS-T system requires water as a medium (a water reservoir), while the piezo system used here only needs gel, thus simplifying set up.

Recently, Divani *et al*. used the same device to show the feasibility of inducing different degrees of ESW-induced TBI^26^. Their rats were divided into two groups receiving different numbers of ESW iterations to the cortex. The authors found that the group receiving more iterations showed greater behavioral deficits, which was consistent with histologic severity. Their histologic samples included H&E-stained sections showing superficial cortical damage, such as hemorrhages and cavities, and immunostained sections showing axonal injury at the site of injury and the underlying white matter. Like Divani’s model, ours was also created using a clinical device and showed different degrees of injury severity. However, our study intravenously injects microbubbles (SonoVue) to enhance the cavitation effect in the brain. Therefore, the severity of our proposed model could be controlled by ESW parameters (intensity and iteration) along with the concentration of microbubbles, providing more control over injury severity. The effects of different concentrations of SonoVue on the brain tissue after ESW exposure have been studied in our previous report^15^. With the use of microbubbles, our model produced injury in the deep brain region (e.g., the striatum), unlike the superficial cortical injury in Divani’s model. In addition, we provide information about progress of apoptotic cell death, neuroinflammation, and gliosis.

The advantages of our method for creating traumatic contusions and ICH can be summarized as follows **1)** It is non-invasive in that it does not require craniotomy. **2)** It is fast and easy to implement, using a commercially available ESW device. **3)** A focal injury can be created and the location, severity and injury type (contusion or ICH) can be adjusted using the different parameters provided by the device. **4)** Results are highly reproducible. **5)** It mimics the barotrauma mechanism as a cause in human traumatic contusion and ICH by adding microbubbles to enhance ESW-induced cavitation.

In addition, the piezo system can be readily used without modification for experiments with larger animals, which may be necessary for some preclinical therapeutic testing. In our proposed model, when ESWs propagate through the rat skull (about 0.75 mm thickness), the loss of the peak pressure is estimated to be approximately 20%. The broad-band nature with low frequency components (audible) of the ESW used allows for deeper penetration in larger animals such as pigs, compared with focused ultrasound systems which usually require craniotomies^27^. Further larger animal studies are needed to confirm this benefit.

### The biophysics behind our model

**Figure 10:**
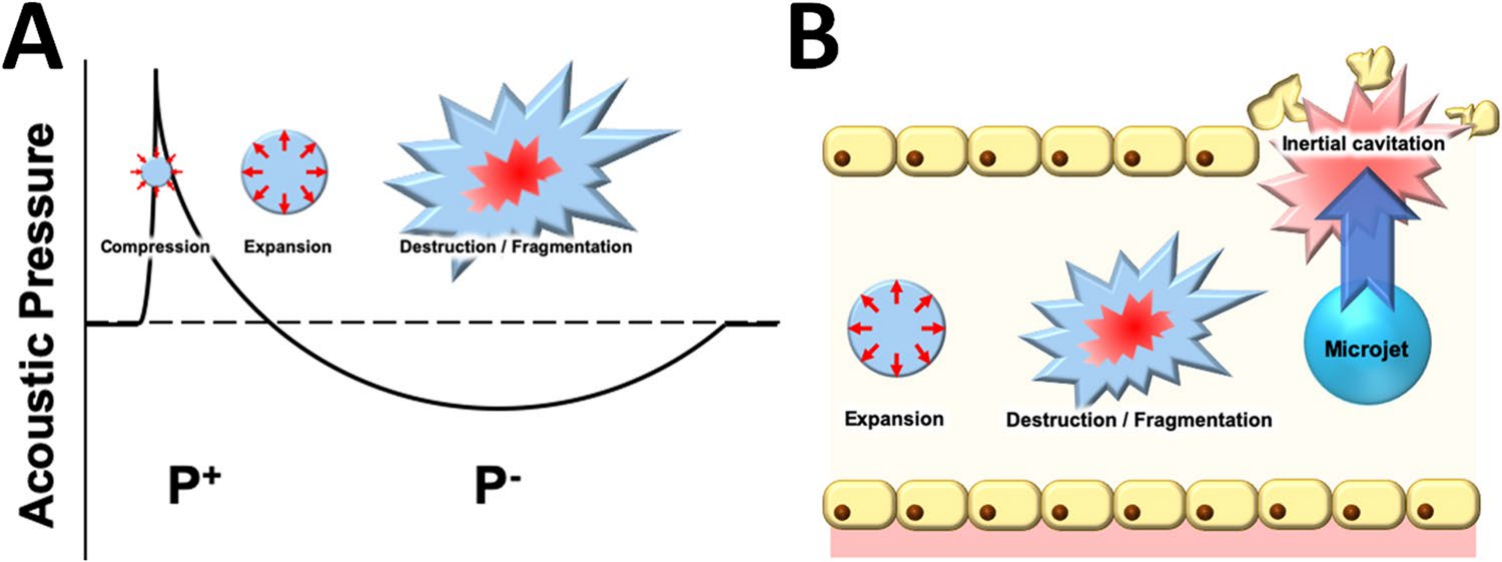
The biophysics behind our model. (**A**) Generation and collapse of the microbubbles under acoustic pressure. P+, positive pressure; P−, negative pressure. (**B**) Microject (a mechanical stress) generation and surrounding tissue damage.

ESW is a strong acoustic wave that creates violent changes in pressure. In medicine, ESW is most commonly used for lithotripsy to treat kidney stones. When ESWs propagate through a fluid (such as water or blood), it’s acoustic pressure induces the formation of microbubbles (i.e. acoustic cavitation) which repeatedly compress and expand, eventually resulting in their violent collapse^28^, as illustrated in Fig. 10A. This process is known as inertial cavitation, and produces strong mechanical stress such as microjets (fluid jets) in the surrounding structure. In brain tissue, microjets can cause blood vessel permeability and even tissue damage^29^, as shown in Fig. 10B.

### Mechanism of injury: ESW-induced cavitation and barotrauma

It has been suggested that the brain lesions found in TBI and ICH patients may share a common underlying mechanism of injury - barotrauma^5^. That is, in both entities, barotrauma from pressure waves propagate through the intracranial contents to cause brain injury. More specifically, the lesions in TBI have been associated with external barotrauma from an impact to the head^5,30^, while some lesions after ICH may result from an internal pressure waves due to the sudden expansion of the hematoma, which immediately propagates through the intracranial contents^5,31^. In terms of pathophysiology, in both TBI and ICH patients, there is an early reduction in the cerebral metabolic rate of oxygen without ischemia or mitochondrial dysfunction, and transient focal increases in regional glucose metabolism a few days after injury, also suggesting the existence of a common underlying barotrauma mechanism^32–34^. We believe that our model at least partially recapitulates this barotrauma, since the applied ESWs came from a probe outside the brain and the acute bleeding was caused by BBB disruption due to an microbubble-enhanced cavitation effects inside the brain. Most of the histopathological changes, cytokine changes and imaging findings from the present study are compatible with those patterns reported in literature^14^. Even the result showing two peaks (acute and delayed) of apoptosis after ESW exposure is compatible with a previously reported TBI model^35^. The delayed activation of microglia was also similar to the reported pattern^36^. Therefore, our model can reliably recapitulate traumatic contusion and ICH in terms of mechanism, pathophysiology, and radiographic and histopathological changes.

Angstman *et al*., who earlier used an off-the-shelf device to establish a non-mammalian model of blast-related brain injury^37,38^, reported possible effects of ESW to induce injury^37^. Based on the mechanisms they proposed, the primary effect of ESWs applied in the present study is the damage caused by components of the ESW itself, that is the brain tissue disruption followed by bleeding. The secondary effects is degraded hematoma-induced cytotoxicity, leading to more cell death and neuroinflammation seen in our model. Angstman *et al*. proposed that the tertiary and quaternary effects are respectively the bodily impact with other objects and crushing injuries from falling objects. In our model, the cavitation effect was substantially enhanced by the addition of microbubbles. The hemorrhagic areas were consistently found at the focal area of our ESW system and thus no significant tertiary or quaternary effects (such as skull damage) were present.

### Two TBI models relevant to traumatic contusion and ICH: the weight drop and CCI models

In weight drop models, the skull or exposed brain is struck by a free-falling guided weight. The Feeney and Shohami models both create focal injury, the severity of which can be controlled by adjusting the mass of the weight and the height from which it falls^39,40^. In Feeney’s weight-drop model, the weight is delivered to the intact dura exposed through craniotomy, and causes a cortical contusion. This model is inexpensive and easy to perform, but relatively high mortality rates and variability of injury severity limited its application. Shohami’s model does not require craniotomy and the device is easy to operate. However, the results are not highly reproducible.

Currently, CCI is the most suitable model for traumatic contusion as it produces a significantly pronounced cortical contusion and subarachnoid hemorrhage^1,2^. It typically uses a device that rapidly accelerates a rod to impact the craniotomy-exposed cortical dural surface. The advantages of the CCI model include the ability to directly control the extent of physical damage and the lack of a rebound injury as seen in the weight drop model, thus significantly reducing mortality. However, the CCI model requires craniotomy and a complex system that needs regular maintenance. Craniotomies have been shown to disrupt normal brain tissue, activate microglia, and stimulate immune response, producing gliosis which indicates brain injury. Animal behavioral data also support that neurological deficits can be detected from craniotomies alone^7^. Our ESW model moots the need for craniotomy and has extremely low mortality.

### Limitations and future work

In the present study, piezo technology provided a stable and convenient source of ESW. However, it is difficult to visualize and measure the pressure profiles due to electromagnetic field noise^24^, which may limit detailed investigations into the relationship between ESW pattern and obtained injuries.

By contrast, using ESW for therapeutic purposes must avoid ESW-induced brain injuries. For example, ESW has been transcranially applied to improve neurological function and stimulate vigilance in patients with unresponsive wakefulness syndrome^41^. For therapeutic purposes, the ESW parameters must be carefully adjusted, since high intensity and frequency will cause brain injury, as described in the present study. Safe brain stimulation may be achieved. Our previous study used the same device but with an intensity level of 9.79 MPa (peak negative pressure), a pulse repetition frequency of 5 Hz, and no microbubbles, to transiently open the BBB after 50 iterations without substantial and irreversible brain tissue damage^15^.

The TBI/ICH-related pathology and pathophysiology are very complex, and the data presented here are basic. Therefore, further study of brain abnormalities after exposure to ESWs derived from piezo technology is needed to apply this model for testing novel therapeutics. For example, strong cavitation has been shown to produce high oxidative stress, eventually leading to brain tissue damage and apoptosis^42^. Hypothermia may be used to reduce oxidative stress and ameliorate potential damage from ESW, as shown in other rat^43^ and *C. elegans* models^38^.

### Conclusion

We demonstrate a non-invasive focal TBI preclinical model that is reliable, fast, easy to use, and capable of producing injuries histologically identical to traumatic contusion and ICH. By adjusting the parameters and focus, we can change the extent and location of contusion and ICH in a highly reproducible manner.

## Data Availability

The authors declare that all data are available.
